# Differences between Human and Mouse IgM Fc Receptor (FcµR)

**DOI:** 10.3390/ijms22137024

**Published:** 2021-06-29

**Authors:** Hiromi Kubagawa, Christopher M. Skopnik, Khlowd Al-Qaisi, Rosaleen A. Calvert, Kazuhito Honjo, Yoshiki Kubagawa, Ruth Teuber, Pedram Mahmoudi Aliabadi, Philipp Enghard, Andreas Radbruch, Brian J. Sutton

**Affiliations:** 1 Deutsches Rheuma-Forschungszentrum, 10117 Berlin, Germany; christopher.skopnik@drfz.de (C.M.S.); kh.kaissius@gmail.com (K.A.-Q.); Ruth.Teuber@drfz.de (R.T.); Pedram.Mahmoudi@drfz.de (P.M.A.); radbruch@drfz.de (A.R.); 2 Randall Centre for Cell and Molecular Biophysics, King’s College, London SE1 1UL, UK; rosy.calvert@kcl.ac.uk (R.A.C.); brian.sutton@kcl.ac.uk (B.J.S.); 3 Department of Pathology of University of Alabama at Birmingham, Birmingham, AL 35294, USA.; khonjo@uab.edu (K.H.); ykubagawa@gmail.com (Y.K.); 4 Department of Nephrology and Medical Intensive Care, Charité-Universitätmedizin, 10117 Berlin, Germany; philipp.enghard@charite.de

**Keywords:** FcR, FcµR, pIgR, Fcα/µR, IgM binding, species difference, 3D structure, computational structural model

## Abstract

Both non-immune “natural” and antigen-induced “immune” IgM are important for protection against pathogens and for regulation of immune responses to self-antigens. Since the bona fide IgM Fc receptor (FcµR) was identified in humans by a functional cloning strategy in 2009, the roles of FcµR in these IgM effector functions have begun to be explored. In this short essay, we describe the differences between human and mouse FcµRs in terms of their identification processes, cellular distributions and ligand binding activities with emphasis on our recent findings from the mutational analysis of human FcµR. We have identified at least three sites of human FcµR, i.e., Asn66 in the CDR2, Lys79 to Arg83 in the DE loop and Asn109 in the CDR3, responsible for its constitutive IgM-ligand binding. Results of computational structural modeling analysis are consistent with these mutational data and a model of the ligand binding, Ig-like domain of human FcµR is proposed. Serendipitously, substitution of Glu41 and Met42 in the CDR1 of human FcµR with mouse equivalents Gln and Leu, either single or more prominently in combination, enhances both the receptor expression and IgM binding. These findings would help in the future development of preventive and therapeutic interventions targeting FcµR.

## 1. Introduction

Two separate lineages of lymphocytes are generated in their distinctive tissue sites and involved in adaptive immunity. B lymphocytes are developed in the bone marrow in mammals and bursa of Fabricius in chickens and contribute humoral immunity, whereas T lymphocytes are generated within the thymus and contribute to cellular immunity [1]. Antibodies or immunoglobulins (Igs), key players in humoral immunity, have dual binding activities to antigens via their N-terminal variable domains in the Fab region and to effector molecules via their C-terminal constant domains in the Fc region. Of the five different antibody classes (IgM, IgD, IgG, IgA and IgE), IgM is the first Ig isotype to appear during phylogeny, ontogeny and immune responses. Two forms of IgM exist that differ in the C-terminus of the µ heavy chain. Alternative splicing with a transmembrane exon (µm) generates monomeric membrane-bound IgM as a B cell receptor for antigen, while that with a secretory exon (µs) generates polymeric IgM secreted by plasma cells as a component of humoral immunity. Both pre-immune “natural” and antigen-induced “immune” IgM are shown to be important for protection against pathogens as well as in regulation of immune responses to self-antigens by analyses of *µs*-ablated mice [2]. A variety of secreted and cell surface proteins, such as complement and various types of Fc receptors (FcRs), are involved in binding the Fc portion of antibody, thereby participating in its effector functions. Classical FcRs for switched Ig isotypes (i.e., FcγRs, high affinity FcεRI, FcαR), the receptor for polymeric IgA and IgM (pIgR), the low affinity FcεRII/CD23, and the FcR for neonatal IgG (FcRn) have thus far extensively been characterized at both genetic and protein levels [3,4,5,6,7,8]. Much of the knowledge gained has been translated to clinical practice. On the other hand, the role of IgM FcR (FcµR) as an effector molecule for IgM antibody has just begun to be explored since its identification in 2009 [9]. As several review articles on FcµR have already been published elsewhere [10,11,12,13,14,15], in this short essay we focus on our recent findings on the mutational analysis of FcµR to explore the molecular basis for differences in IgM binding observed in human and mouse FcµRs.

## 2. Differences between Human and Mouse FcµRs

### 2.1. Identification of FcµR cDNAs, Functional Cloning vs Database Search

**Figure 1:**
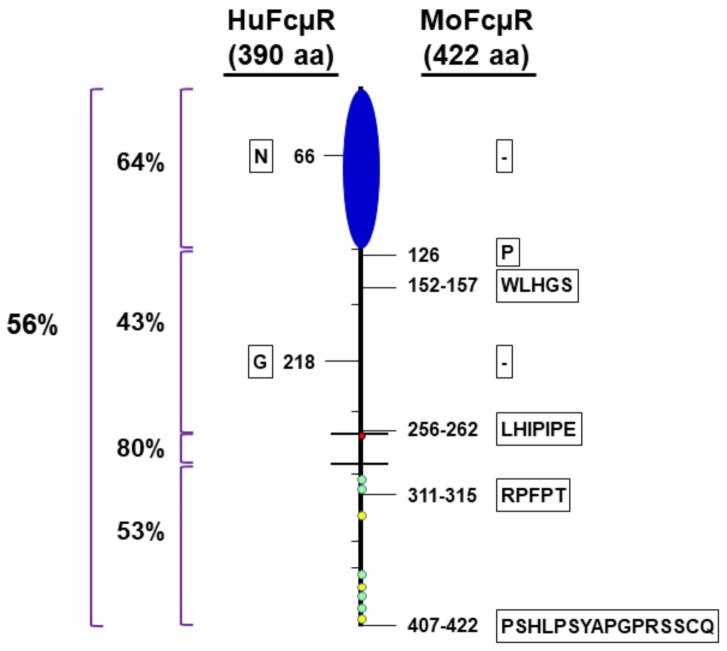
Schematic presentation of homology between human and mouse FcµRs. FcµR is depicted as a racquet-like shape consisting of N-terminal Ig-like domain (blue closed oval shape), stalk region (above the top line), transmembrane (between the two lines) and the cytoplasmic tail (below the bottom line). Hatch marks indicate exon boundaries and small red, green and yellow circles indicate a charged His residue in the transmembrane region and conserved five Ser and three Tyr residues in the cytoplasmic tail, respectively. Numbers on the left indicate percentage identity between human and mouse receptors in the overall or indicated regions. The position of aa addition (single letter code within frame) or gap (- within frame) in human (390-aa, left) and mouse (422-aa, right) FcµR are shown beside the cartoon.

Historically, IgM binding to many different hematopoietic cell types including myeloid cells has been reported in both humans and rodents, first by electron microscopy (EM) using antibody and the corresponding radiolabeled antigen, then by rosette formation with IgM antibody-coated erythrocytes and finally by flow cytometry using fluorochrome-labeled IgM (see refs in ref. [9]). The molecular identity of the receptor, however, was obscure until 2009, when the functional cloning strategy (i.e., IgM binding) was employed for the cDNA libraries of two human cell types that are known to bind the Fc portion of IgM molecules, namely, phorbol myristate acetate (PMA)-activated 697 pre-B cell line and chronic lymphocytic leukemia (CLL) B cells [9]. The resultant FcµR cDNA encodes a type I transmembrane protein consisting of an Ig-like domain at the N-terminus, the remaining structurally uncharacterized extracellular region (called stalk region), a transmembrane (TM) segment containing a charged His residue, and a relatively long cytoplasmic (CY) tail containing conserved five Ser and three Tyr residues. Ironically, the FcµR cDNA turned out to be identical to that of TOSO or Fas apoptosis inhibitor molecule 3, which was also functionally cloned as a strong inhibitor of Fas-mediated apoptosis induced by agonistic IgM anti-Fas monoclonal antibody (mAb) [16]. There is now a consensus of nomenclature of this gene as *FCMR* (for humans) and *Fcmr* (for other species) [17]. The mouse orthologue was then identified by basic local alignment search technique database analysis. Unique structural characteristics, such as lack of N-linked glycosylation sites and presence of the charged His residue in the TM region, as well as of the conserved Ser and Tyr residues in the CY tail, were preserved. However, the overall amino acid (aa) identity between the 390-aa human and 422-aa mouse FcµRs is low (~56%). The degree of homology in each segment is in order: TM (80%) > Ig-like domain (64%) > CY (53%) > stalk (43%). The mouse receptor has insertions of 1–16 aa in the stalk and CY regions and a single aa deletion in each of the Ig-like and stalk regions (Figure 1).

### 2.2. Cellular Distribution, Lymphocytes vs Only B Cells

In addition to low homology, another clear difference is the cellular distribution of FcµR in these two species. FcµR in humans is expressed by B, T and, to a lesser extent, NK cells, whereas FcµR in mice is expressed by B cells only [9,18,19,20,21]. While the functional roles of FcμR in murine non-B cell populations have been shown by comparison between *Fcmr* deficient (KO) and wild-type (WT) mice [22,23,24,25,26,27], direct evidence that FcµR is indeed expressed on the surface of non-B cells seems to be lacking at least to four authors (H.K., C.M.S., K.H., and Y.K.). The lymphocyte-restricted distribution of FcµR is thus quite distinct from the distribution of FcRs for switched Ig isotypes (i.e., FcγRs, FcεRs, FcαR (only in humans)), which are expressed by a variety of hematopoietic and non-hematopoietic cells and function as central mediators coupling innate and adaptive immune responses [28]. It is thus reasonably assumed that the FcµR on lymphocytes may have a distinct function from other FcRs [15].

Notably, the detection of human FcµR on freshly prepared lymphocytes can be achieved by both receptor-specific mAbs and IgM ligands, albeit more sensitive for the former than the latter, but pre-incubation of lymphocytes in IgM-free media for a short time period is required for detection of cell surface FcµR, especially for T cells [9]. By contrast, in the case of mouse B cells, FcµR is clearly demonstrable on their cell surface by receptor-specific mAbs*, but hardly detectable by its IgM binding [20]. Several possibilities might account for difficulty in the detection of FcµR on B cells with IgM ligands. These include (i) blockage of the ligand binding site with endogenous IgM, although the IgM-bound FcµR must be rapidly internalized, (ii) cleavage of the ligand-binding Ig-like domain by endogenous proteases, and (iii) conformational inaccessibility of the Ig-like domain to bind IgM ligands. (*We made a panel of 10 different mAbs, but curiously none of them recognized an epitope located in the Ig-like domain of mouse FcµR. The results differed significantly from those of human FcµR-specific mAbs: three were specific for the Ig-like domain and seven for the stalk region.) Other groups, including ours, also had difficulty in demonstrating clear-cut IgM binding by mouse FcµR, unlike the human receptor. We found clear IgM binding by marginal zone B cells from *µs-*ablated mice (K.H. and H.K., unpublished). The only convincing evidence for IgM binding by freshly prepared B cells came from the adoptive transfer experiments where control or B cell-specifically *Fcmr-*deleted, IgM^b^-expressing B cells were transferred into congenic IgM^a^-expressing mice. At day 3 post-transfer, transferred CD19^+^IgD^b^ B cells from *Fcmr-*deleted mice exhibited significantly reduced host-derived IgM^a^ binding in vivo as compared with those from control mice [29]. Collectively, these findings suggest different modes of the ligand binding of FcμR in humans and mice, possibly due to unique post-translational modifications for mouse receptor.

### 2.3. IgM Ligand Binding, Constitutive vs Transient Binding

**Figure 2:**
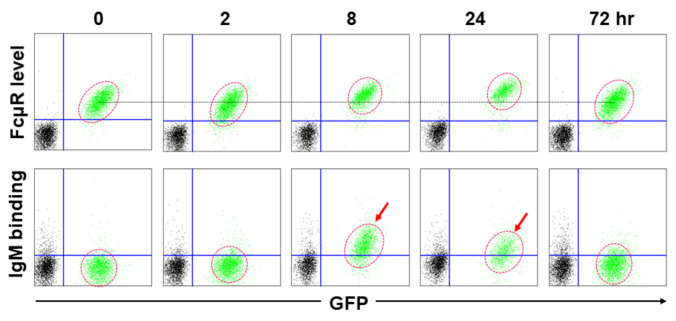
Transient IgM binding by mouse FcµR-bearing transductant. AKR-derived thymoma line BW5147 cells stably expressing mouse FcµR and GFP (green) and WT BW5147 cells (black) were plated at 5 × 10^4^ cells/mL, cultured at 37 °C, and harvested after the indicated time periods. An equal mixture of FcµR^+^/GFP^+^ cells and WT cells was incubated with mouse IgG1κ mAb specific for mouse FcµR (MM3 clone; upper panel) or mouse IgMκ paraprotein (TEPC183; lower panel), washed, and developed by PE-labeled, rat IgG1κ anti-mouse κ mAb (187.1 clone) to determine the FcµR level and IgM binding, respectively. Stained cells were analyzed by flow cytometry. Note the IgM binding by FcµR^+^/GFP^+^ cells at early stages of culture (see arrows) compared to the minimal changes in cell surface receptor levels. GFP^+^ cells are circled with red dotted lines and a black line corresponds with mean fluorescence intensity of PE of FcµR^+^/GFP^+^ cells at 0 h for comparison.

The clearest distinction between human and mouse FcµRs is their ligand binding capability. This was first noted when FcµR-negative, AKR mouse-derived thymoma line BW5147 cells, were stably transduced by each cDNA (along with GFP cDNA) and examined for their IgM-ligand binding. As expected, human FcµR^+^ transductant bound to IgM during the culture period irrespective of its cell growth stages (*constitutive* binding). By contrast, mouse FcµR^+^ transductant bound to IgM only prior to the early cell growth stage, although the receptor levels as determined by receptor-specific mAbs did not significantly alter during cell culture (*transient* binding) (Figure 2). HeLa cell line transiently expressing mouse FcµR exhibited weak binding to IgM [19]. Unlike the above BW5147 transductants, another mouse FcµR^+^ BW5147 transductant exhibited clear binding with mouse polyclonal IgM antibodies against sheep erythrocytes [30]. Similar weak IgM binding results were also obtained with murine B cell lines (A20 and CH31) expressing endogenous FcµR. Treatment of these B cell lines with PMA or pre-incubation with stalk region-specific divalent mAbs enhanced their IgM binding, whereas lipopolysaccharide (LPS) stimulation did not. This suggested an involvement of protein kinase C (PKC), a family of serine/threonine protein kinases, or receptor configuration in the ligand binding, but not of myeloid differentiation primary response 88 (MYD88). It remains unclear what post-translational modification(s) is/are responsible for this transient IgM binding by mouse FcµR.

### 2.4. Dependence of the Ig-Like Domain of FcµR

**Figure 3:**
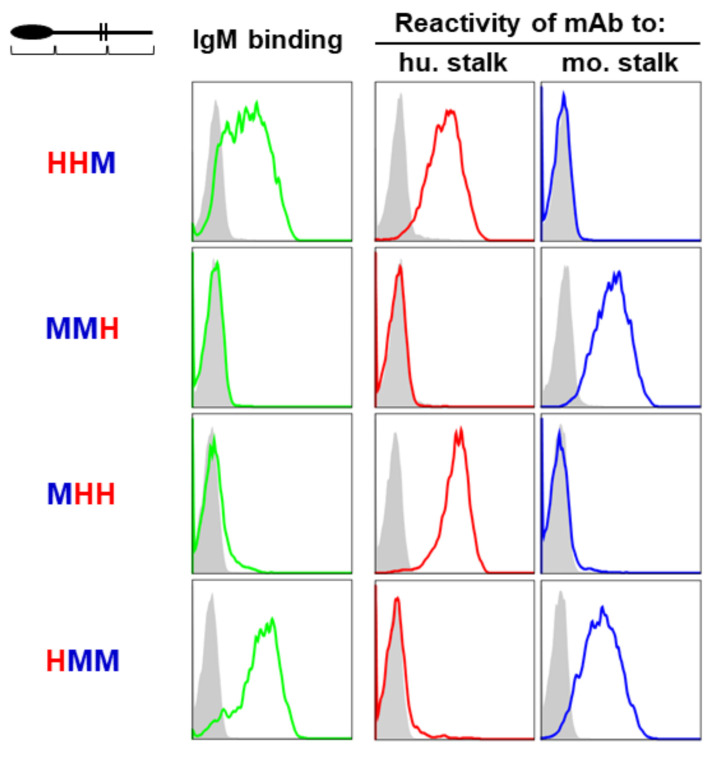
IgM binding of human/mouse chimeric FcµRs. BW5147 cells stably expressing FcµR composed of Ig-like domain, stalk region and transmembrane/cytoplasmic tail (top left) of either human (H) or mouse (M) origin (left) were incubated with TEPC183 IgMκ for IgM binding (green lines), HM14 mAb to the stalk region of human FcµR (hu. Stalk; red lines) or MM3 mAb to the stalk region of mouse FcµR (mo. Stalk; blue lines), before developing with PE-labeled goat anti-mouse Ig antibodies and analyzing by flow cytometry. Note that IgM binding was only observed with chimeric receptor containing human-derived Ig-like domain.

Given the low homology in the stalk and CY regions between human and mouse FcµRs (43% and 53%, respectively), we initially thought that either the stalk or CY region of receptor or both might indirectly influence the IgM-ligand binding. In order to test this idea, each segment of FcµR (i.e., Ig-like domain, stalk/TM, and CY regions) was swapped between human and mouse receptors, before establishing transductants stably expressing chimeric FcµR proteins on their cell surface. (The precise experimental procedures are described in the Tables S1 and S2, and Figure S1) [9,20,31]. The acronym “HHM” indicates the FcµR consisting of the Ig-like domain and stalk/TM region of human origin “H” and the CY region of mouse origin “M”. Cells expressing comparable levels of GFP were enriched from each transductant by FACS and were assessed for their IgM binding activity as well as for their FcµR levels by flow cytometry using a mouse IgMκ paraprotein (TEPC 183) and stalk-region specific mAbs (HM14 and MM3 for human and mouse FcµR), respectively. IgM binding was only observed with chimeric receptors when the Ig-like domain was derived from the human receptor (e.g., HHM and HMM) (Figure 3). Chimeric receptors with the Ig-like domain of mouse origin did not exhibit IgM binding irrespective of human-derived other parts of the receptor (e.g., MHH and MMH). Only subtle changes in IgM binding were observed with the MHH chimeric receptor, suggesting that the contribution of either stalk/TM or CY part of the human receptor, if any, was less important than the Ig-like domain of the human receptor. Thus, contrary to our initial prediction, these findings suggest that the difference in IgM binding observed between human and mouse FcµRs is directly attributed to the ligand-binding Ig-like domain rather than other parts of the receptor.

## 3. Mutational Analysis of FcµR

### 3.1. Amino Acid Sequence Alignment of the Ig-Like Domain of Human and Mouse FcµRs

**Figure 4:**
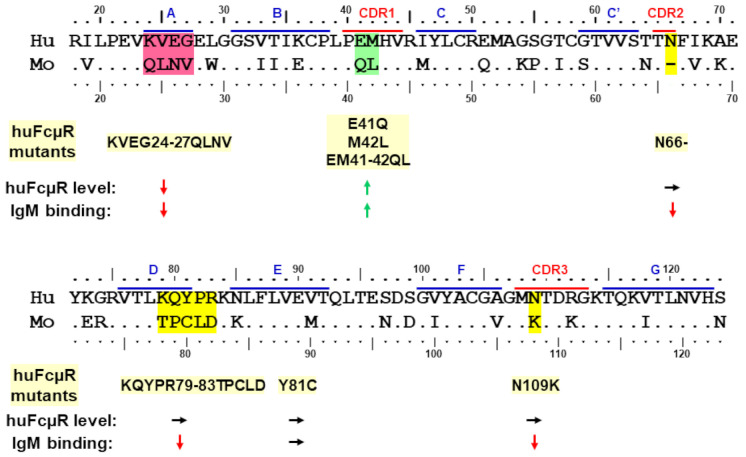
Amino acid sequence alignment of the Ig-like domains of human and mouse FcµRs along with mutational results. The numbers on the top and bottom of the sequence indicate the amino acid (single letter code) position with regard to the first Met residue of human (Hu) and mouse (Mo) FcµR. Amino acid identity and gaps are indicated by dots (•) and dashes (-), respectively. Predicted ß-strands (blue lines) and complementary determining regions (CDRs; red lines) of human FcµR are indicated at the top of the sequence. The human aa residues highlighted in red, green or yellow colors were replaced with the corresponding mouse residues, and eight different human FcµR mutants are shown underneath with pale yellow highlights. Receptor level and IgM binding potential of each human FcµR mutant are indicated as arrows with decrease (↓), increase (↑) or no change (→). Accession numbers of human and mouse FcµRs in NCBI are NM_005449 and NM_026976, respectively.

To determine potentially critical regions responsible for such species differences in ligand binding, the aa sequence of the Ig-like domain of human and mouse FcµRs were aligned with each other based on the secondary structure of human pIgR domain 1 (D1) determined by crystallography [32,33]. As aforementioned, the sequence homology of Ig-like domains between human and mouse FcµRs was 64%. There are several aa sequence differences localized around the putative ligand-binding complementary determining regions (CDRs) that could explain the constitutive and transient IgM binding difference between human and mouse FcµRs (Figure 4). For example, position 41 in CDR1 is a negatively charged Glu in the human receptor but the non-charged Gln in mouse (E vs. Q) and the next position 42 is a nonpolar residue in both species, Met and Leu. It is unclear how these two consecutive differences (EM vs. QL) out of potential five aa residues in the CDR1 affect their ligand binding activities. In this regard, the length of CDR1 is quite different from two other IgM-binding receptors, pIgR and FcR for polymeric IgA and IgM (Fcα/µR). FcµR, which exclusively binds IgM, has five aa residues in CDR1 for many mammals, whereas pIgR and Fcα/µR, which promiscuously binds polymeric IgA and IgM, have nine aa residues [9,11]. It is thus possible that the above two consecutive differences in the CDR1 of human vs mouse FcµR may greatly affect their IgM ligand binding.

Unlike the CDR2 of Ig VH and VL regions, the FcµR CDR2 loop is very short in many mammals, consisting of only two aa residues as in pIgR and Fcα/µR [32,33]. The residue at the position 66 is a non-charged Asn in humans but missing in mice (N vs. -). It is thus conceivable that, if one of the two residues is removed from human FcµR CDR2 (N66-) as in mouse FcµR, then such a deletion mutant may profoundly affect its IgM binding activity. For FcµR CDR3, position 109 is Asn in humans but a positively charged Lys in mice (N vs. K). Position 112 is a positively charged aa residue in both species, Arg and Lys, thereby less likely to contribute to such differential IgM binding in these two species.

In addition to these differences in CDR regions, a stretch of four aa residues at positions 24–27 in the presumed A strand of FcµR are significantly different from each other (KVEG vs. QLNV). The positively charged Lys at position 24 and negatively charged Glu at position 26 in the human receptor are both uncharged Gln and Asn in mice, respectively (K vs. Q and E vs. N). Another distinguishing stretch is five aa residues at positions 79–83 (KQYPR vs. TPCLD) corresponding to the DE loop of FcµR, directly adjacent to CDR2. The changes from human to mouse receptors include a positively charged Lys at position 79 to a non-charged Thr (K vs. T), another positively charged Arg at position 83 to a negatively charged Asp (R vs. D), and an aromatic Tyr at position 81 to a sulfur-containing Cys (Y vs. C) as well as different positions of Pro (82 vs. 79). It is highly likely that these four or five consecutive changes may account for the distinctive IgM binding properties between human and mouse FcµRs.

### 3.2. Site-Directed Mutagenesis

Based on this sequence comparison, we have hypothesized that, if the above different aa residues in human FcµR are replaced by the corresponding mouse residues, then the resultant huFcµR mutants may no longer constitutively bind IgM ligands, similar to mouse FcµR. We have thus made BW5147 transductants, stably expressing eight different substitutions as follows: KVEG24-27QLNV, E41Q, M42L, EM41-42QL, N66-, KQYPR79-83TPCLD, Y81C, and N109K. In addition to these human FcµR replacement mutants, transductants stably expressing human or mouse WT FcµR and only GFP (empty vector) were also prepared as controls. After enriching cells with equivalent levels of GFP for each transductant by FACS, cell surface receptor levels and IgM binding activities of human FcµR WT and mutants were assessed by flow cytometry using two different human FcµR-specific mAbs (ligand binding site-specific HM7 and stalk region-specific HM14) and IgM ligands (of both human and mouse origins). It was noteworthy that none of the above replacements caused significant alterations in the main-chain conformation of the receptor as determined by modeling of the substitutions or changes in the melting temperature assessed by molecular dynamics simulations. The results of receptor levels and IgM binding potential are summarized in a cartoon fashion in Figure 4, and the detailed flow cytometric data have been reported elsewhere [34].

#### 3.2.1. Asn66 in CDR2, Lys79-Arg83 in DE Loop and Asn109 in CDR3

In three human FcµR mutants (N66-, KQYPR79-83TPCLD, and N109K), their receptor levels as judged by HM14 mAb reactivity were comparable to those of human FcµR WT, but their ligand binding activities with both human and mouse IgM preparations were significantly diminished (Figure 4). This suggests that these three sites (Asn66, Lys79-Arg83 and Asn109) are responsible for the constitutive IgM binding of human FcµR, and the computational structural modeling analysis (see below) revealed that they were located in CDR1, DE loop and CDR3, respectively. In the N66- and KQYPR79-83TPCLD mutants, both HM14 and HM7 mAbs exhibited essentially similar reactivity, whereas in the N109K mutant, the HM7 mAb reactivity, unlike HM14, was significantly reduced. This implied that the epitope recognized by HM7 mAb, which was predicted to be near the ligand-binding site of human FcµR, might be susceptible to a point mutation of N to K at position 109. A single aa replacement mutant Y81C, unlike the five consecutive replacements of Lys79-Arg83, exhibited comparable cell surface receptor level and IgM binding activity as human FcµR WT. The unaffected findings for human FcµR Y81C mutant were unexpected, because we initially thought that this solvent-exposed, additional free Cys residue at position 80 of mouse FcµR might account for its transient ligand binding activity. Nevertheless, the finding that the five consecutive substitutions KQYPR79-83TPCLD, but not a single replacement Y81C, affected IgM-ligand binding without alteration of receptor levels was evident. Collectively, these findings suggest that at least three sites, namely, N66 in CDR2, K79-R83 in DE loop, and N109 in CDR3 in human FcµR, are essential for its constitutive IgM binding.

It should be noted that in our subsequent analysis when all the above three sites of human residues (N66, K79-R83, and N109) were used to replace the mouse equivalents, the resultant mouse FcµR triple mutant (-66N, TPCLD78-82KQYPR, K108N) was found to constitutively bind IgM, like human FcµR (R.T., et al., unpublished observation). This finding with the mouse revertant strongly supports that the aforementioned three sites are indeed critical for IgM binding in human FcµR.

#### 3.2.2. Glu41 and Met42 in CDR1

Unlike the above four human FcµR mutants (N66-, KQYPR79-83TPCLD, Y81C, and N109K) which did not affect cell surface expression of FcµR as defined by the HM14 mAb reactivity, three CDR1 mutants (E41Q, M42L, and EM41-42QL) showed significant increases in both surface receptor levels and IgM binding activities in the order: EM41-42QL >> E41Q > M42L (Figure 4). These increases were observed with both HM14 and HM7 mAbs and with both human and mouse IgM ligands. The enhancement of particularly IgM binding activity by introducing mouse Gln and Leu residues into human corresponding sites was an unexpected finding, because the mouse FcµR had limited or transient IgM binding activity as compared with human FcµR. The molecular basis for this enhancement remains to be elucidated, but several possibilities including tissue transglutaminase (tTG)-mediated modification of the Gln41 in mouse FcµR may be considered. In this regard, tTG-mediated cross-linkage occurring between the γ-carboxamide group of a Gln residue and the ε-amino group of a Lys residue to form an ε-(γ-glutamyl) lysine iso-peptide bond is known to alter the molecular masses and functions of membrane proteins [35].

#### 3.2.3. Lys24-Gly27 in A Strand

Another human FcµR mutant KVEG24-27QLNV exhibited significant reduction of cell surface receptor levels defined by the reactivity with both HM14 and HM7 mAbs, as well as diminished binding activity with both human and mouse IgM ligands (Figure 4). The four aa stretch from Lys24 to Gly27 was located in the presumed A strand of human FcµR and this N-terminal region could be essential for maintaining the proper structure of the receptor expressed on the plasma membrane, although the results from modeling of this mutation, and the melting temperature assessed by molecular dynamics simulations, suggested no significant alteration in the main conformation of the receptor.

Taken together, several remarkable features of the human FcµR were revealed from the mutational analysis. First, at least three sites of human FcµR, namely two Asn residues at positions 66 in CDR2 and 109 in CDR3 and a stretch of five aa from Lys79 to Arg83 in the DE loop, were essential for its constitutive ligand binding potential. Second, replacement of Glu41 and Met42 in CDR1 with the corresponding mouse residues Gln and Leu resulted in enhancement of both cell surface receptor levels as well as IgM-ligand binding activity. Third, the four aa stretch of Lys24 to Gly27 in the A strand of human FcµR might be critical for maintenance of its proper structure on the plasma membrane as this replacement mutant exhibited marked reduction of both receptor levels and IgM binding.

## 4. Structural Aspects of FcµR and IgM

### 4.1. Computational Structural Modeling of Human FcµR

**Figure 5:**
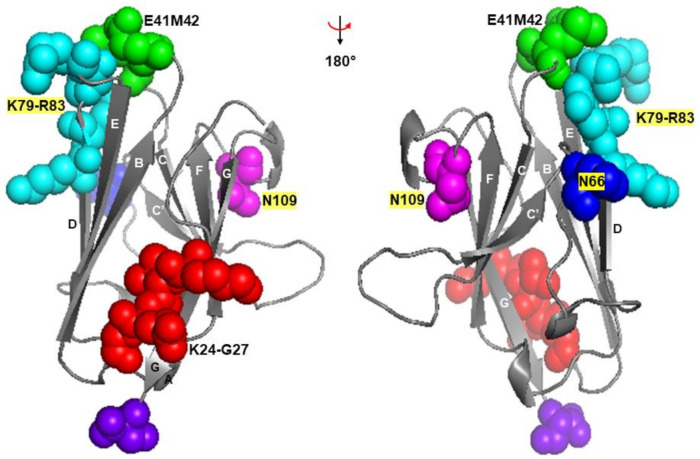
Model structure of human FcµR Ig-like domain. Models of human FcµR domain (left) and after 180° horizontal rotation (right) are shown highlighting the aa residues mutated in this study: K24-G27 (red), E41/M42 (green), N66 (blue), K79-R83 (cyan), and N109 (magenta). The C-terminus (purple) and β strands are also indicated. Polymeric Ig receptor domain 1 (PDB 5D4K) was used as a template to create the human FcµR model.

Although the precise mode of binding of FcµR with IgM ligand must await the crystal structure analysis of FcµR and FcµR/IgM complex, the crystal structure of pIgR D1 (or secretory component (SC)), which shares ~31% aa sequence identity with the Ig-like domain of human FcµR, was fortunately solved [32,33]. Many core features are conserved between FcµR and pIgR D1, including two intra-chain disulfide bonds (Cys37-Cys104 and Cys49-Cys58) and a salt bridge between Arg75 and Asp98 of human FcµR. Thus, we performed a computational structural analysis by using pIgR D1 (PDB 5D4K) as a template to create a structural model of the Ig-like domain of human FcµR WT. We then created models of human FcµR with substitutions of mouse aa residues as described above (including the deletion at position 66 (N66-)). None of the substitutions or the deletion caused significant changes to the main-chain conformation of the Ig-like domain of FcµR. The model of FcµR Ig-like domain is shown in Figure 5, indicating Lys24-Gly27 in A strand, Glu41/Met42 in CDR1, Asn66 in CDR2, Lys79-Arg83 in DE loop, and Asn109 in CDR3. This showed that the CDR1 Glu41/Met42 as well as the CDR2 Asn66 and the DE loop Lys79-Arg83 were all exposed and close to one another in three dimensional space. On the other hand, the CDR3 Asn109 was tilted away from the CDR1 and CDR2 and the BED sheet. This feature was similar to pIgR D1 but different from Ig VH and VL domains [32,36]. Furthermore, another feature that the CDR3 loop of pIgR D1 is stabilized by hydrogen bonds between Asn115 within the loop and two residues (Arg52 in the C strand and Thr66 in the C’ strand) [32], was also preserved in human FcµR based on computational modeling. Collectively, the predicted model of the structure of human FcµR ligand-binding domain supports the mutational data, but the precise interaction of FcµR with IgM ligands as well as the different modes of binding of FcµR and pIgR with their ligands (IgM vs. polymeric IgA and IgM) must await crystal structure analysis.

### 4.2. Recent Structural Aspects of IgM Ligand

The binding of pentameric IgM with human WT FcµR^+^ transductant was inhibited by addition of its Fc_5_µ fragments consisting mostly of Cµ3/Cµ4 domains in a dose dependent manner, but not of its Fabµ fragments, thereby defining the IgM FcR [9]. Subsequent results from domain swapping experiments by Lloyd et al. indicated that the Cµ4 domain contributed more to binding with FcµR than the Cµ2 and Cµ3 domains [37]. They also employed all-atom molecular dynamics simulations and inspected the potential interface of the Cµ2-Cµ4 of IgM and the human FcµR Ig-like domain. Several polar and hydrophobic residues were predicted to be involved in the interaction. These included extensive hydrogen-bonding between side chains and backbone atoms involving Pro321 in Cµ3 and His327, Leu420 and Asn422 in Cµ4 of IgM, and Pro40 and Glu41 in the CDR1 and Arg83 and Asn85 in the DE loop of FcµR, surrounding a hydrophobic pocket consisting of Gly323, Ala325, Leu326 and Ala419 in Cµ4, and Met42 and Val44 in the CDR1 and Pro82 in the DE loop of FcµR [37]. (The numbers in IgM indicate the aa position from the first N-terminal aa residue in the Cµ1 domain, not from the first Met residue that was applied for FcµR, because of variable lengths of Ig VH regions.) On the other hand, our docking model of pentameric IgM and FcµR revealed that Gln387 in the Cµ4 and to a lesser extent, Glu275 in the Cµ3 of IgM and Asn66 in the CDR2, Arg83 in the DE loop and Asn109 in the CDR3 of FcµR were all at the interface, suggesting their potential involvement in IgM binding [34]. This model was thus consistent with substantial loss of IgM binding in mutants involving removal of Asn66 or substitution of Lys79-Arg83 or Asn109 with the mouse equivalents and with the involvement of both Cµ4 and Cµ3 domains in FcµR binding [9,37,38,39,40]. Intriguingly, we did not see contact of Glu41 and Met42 in the CDR1 of FcµR with any part of IgM Fc.

As a result of recent technical advances in structural biology, IgM has received a significant revision in terms of its structure (see reviews [6,13,41]). IgM was traditionally considered as a symmetrical pentamer like a planar star-shaped pentagon, with the Fab fragments pointing away from the inner core of the Fc regions. However, results from the recent EM structures indicate that IgM is an asymmetric pentamer, resembling a hexagon with a missing triangular segment where several proteins fit. They include the J chain, the pIgR SC and the apoptosis inhibitor of macrophages (AIM; also called CD5-like antigen or soluble protein α (Spα)) [42,43]. Unlike these IgM-binding proteins, our docking model suggested that FcµR did not fit in this “gap” region, but instead, interacted with the Cµ4 and to a lesser extent, the Cµ3 domains of each monomeric subunit of the human IgM pentamer. This was consistent with the findings that FcµR, unlike pIgR, could interact with both J chain-containing pentameric and J chain-lacking hexametric IgM with similar affinities [44]. Thus, we predict that FcµR binds IgM in a different fashion to pIgR.

## 5. Conclusions

While both human and mouse FcµR genes are located in a syntenic region of chromosome 1 adjacent to two other IgM-binding receptor genes, pIgR and Fcα/µR, we have described clear distinctions of these two receptors with regard to the identification process, cellular distribution and ligand binding potential. By taking advantage of the IgM-binding difference between human (constitutive) and mouse (transient) FcµR, we could identify three critical sites in IgM binding of human FcµR: Asn66 in the CDR2, Lys79 to Arg83 in the DE loop and Asn109 in the CDR3. Results from computational structural modeling analysis supported these mutational data. Importantly, when these aa residues were used to replace the corresponding mouse residues, the resultant mouse FcµR triple mutant constitutively bound to IgM like the human receptor. This indicated that the above three sites were indeed essential for the constitutive ligand binding of human FcµR. Serendipitously, substitution of Glu41 and Met42 in the CDR1 of the human FcµR with the mouse equivalents Gln and Leu, either singly or more prominently in combination, enhanced both receptor expression and IgM binding. These findings would help in future development of preventive and therapeutic interventions targeting FcµR.

## Supplementary Materials

The following are available online at https://www.mdpi.com/article/10.3390/ijms22137024/s1: Table S1: Primers for overlap extension PCR for human/mouse chimeric FcµR constructs. Table S2: Strategy for overlap extension PCR for human/mouse chimeric FcµRs. Figure S1: Strategy for overlap extension PCR for human/mouse chimeric FcµR constructs.
