# Supplementary material for: Differences between Human and Mouse IgM Fc Receptor (FcµR)
Source: Int J Mol Sci. 2021 Jun 29;22(13):7024. doi: 10.3390/ijms22137024 (PMC8267714; doi:10.3390/ijms22137024)
Supplement: Supplementary file 1 [file ijms-22-07024-s001.zip › ijms-1261244-supplementary.pdf]

Supplementary Material

Materials and Methods

Preparation of human/mouse chimeric FcμR constructs: Swapping of each functional domain [*i.e.*, Ig-like (Ig-D), stalk/transmembrane (ST/TM), cytoplasmic (CY)] between human and mouse FcμRs was performed by overlap extension PCR (1) using primers flanking at 3′ site with sequences corresponding to either 5′ or 3′ side of each domain of the other species (see Fig. S1). Nucleotide sequences of forward (F) and reverse (R) primers used (1-12) are listed in Table S1 corresponding with human FcμR (red) and mouse FcμR (blue), identical in both species (purple), and restriction enzyme sites (black italics), along with their lengths (-mer) and melting temperature (Tm). Initial PCR amplifications (1-1 to 1-4 and 3-1 to 3-4) were performed using human or mouse FcμR cDNA as a template with a set of indicated primers, AccuTaq LA DNA polymerase (Invitrogen) or PrimeSTAR DNA polymerase (Takara) and cycle conditions (see Table S2 and Fig. S1). Each amplified PCR product of the expected size was gel purified and mixed in an equimolar ratio as template DNAs (1-1/1-2, 1-3/1-4, 3-1/3-2 and 3-3/3-4) for the subsequent overlap extension PCR (2-1, 2-2, 4-1 and 4-2, respectively). The final PCR products were subcloned into pCR-Blunt vector, cut with *Eco*RI and *Not*I, ligated into the pMXsPIE retroviral vector that contains a GFP cDNA and *Streptomyces alboniger* puromycin-N-acetyltransferase cDNA. After confirming sequence identity, the resultant chimeric FcμR cDNAs were transduced in the BW5147 T cell line and GFP$^+$ cells were enriched by FACS and in the presence of puromycin (1 μg/mL) as described previously (2,3).

Table S1. Primers for overlap extension PCR for human/mouse chimeric FcμR constructs

Primer	Sequence	Discription	-mer	Tm
1	5'-*gaattc* TAGAAGGGACAATGGACTTC-3'	*Eco*RI-5'UTR-ATG-huFcμR SS (F)	26	58
2	5'-ATGTCCATAATGAATACGAGCCATCATGGGAAG-3'	moFcμR Ig/hu FcμR ST (F)	45	68
3	5'-AAAGGAGGAGAGCCCTCTCCAGGCGGGCCCGC-3'	moFcμR TM/huFcμR CY (F)	32	74
4	5'-GTTCTGGGTATTCACTGTGGACATTCAGGGTG-3'	moFcμR ST/huFcμR Ig (R)	32	62
5	5'-GTCTGGAGGAGGCTTTCCTCCTTTCAACGGCC-3'	moFcμR CY/huFcμR TM (R)	32	62
6	5'-*gcggccgc* TCAGGCAGGAACATTGATGTAG-3'	*Not*I-stop-huFcμR CY (R)	30	64
7	5'-*gaattc* AGGGAACCATGGACTTTTGG-3'	*Eco*RI-5'UTR-ATG-moFcμR SS (F)	26	60
8	5'-ATGTCCACAGTGAATACCCAGAACCATTCTGG-3'	huFcμR Ig/moFcμR ST (F)	32	58
9	5'-AAAGGAGGAAAGCCTCCTCCAGACGTGCGGGC-3'	huFcμR TM/moFcμR CY (F)	32	70
10	5'-ATGGCTCGTATTCATTATGGACATTCAGGGTG-3'	huFcμR ST/moFcμR Ig (R)	32	58
11	5'-GCCTGGAGAGGGCTCTCCTCCTTTGAATGGCT-3'	huFcμR CY/moFcμR TM (R)	32	60
12	5'-*gcggccgc* TCATTGGCATGAAGATCTG-3'	*Not*I·stop·moFcμR CY (R)	27	54

Table S2. Strategy for overlap extension PCR for human/mouse chimeric FcμRs

PCR	Primers 5'	Primers 3'	Template DNA	bp	Amplified products Ig	Amplified products ST/TM	Amplified products CY	Amplification cycles: denat.	Amplification cycles: anneal.	Amplification cycles: exten.	
1-1	1 (58°)	5 (62°)	huFcμR	870	H	H		0:30 (94°)	0:30 (52°)	2:00 (68°)	x35*
1-2	9 (70°)	12 (54°)	moFcμR	414			M	0:30 (94°)	0:30 (52°)	1:00 (68°)	
1-3	7 (60°)	11 (60°)	moFcμR	900	M	M		0:30 (94°)	0:30 (58°)	2:00 (68°)	
1-4	3 (74°)	6 (64°)	huFcμR	352			H	0:30 (94°)	0:30 (62°)	1:00 (72°)	
**2-1**	1 (58°)	12 (54°)	PCR 1-1/PCR 1-2	1261	H	H	M	0:30 (94°)	0:30 (52°)	2:00 (68°)	
**2-2**	7 (60°)	6 (64°)	PCR 1-3/PCR 1-4	1228	M	M	H				
3-1	7 (60°)	10 (58°)	moFcμR	396	M			0:10 (94°)	0.10 (56°)	1:00 (72°)	x30**
3-2	2 (68°)	6 (64°)	huFcμR	820		H	H	0:10 (94°)	0:10 (59°)	2:00 (72°)	
3-3	1 (58°)	4 (62°)	huFcμR	402	H			0:10 (94°)	0:10 (56°)	1:00 (72°)	
3-4	8 (58°)	12 (54°)	moFcμR	919		M	M	0:10 (94°)	0:15 (52°)	2:00 (72°)	
**4-1**	7 (60°)	6 (64°)	PCR 3-1/PCR 3-2	1192	M	H	H	0:10 (94°)	0:10 (58°)	2:00 (72°)	
**4-2**	1 (58°)	12 (54°)	PCR 3-3/PCR 3-4	1297	H	M	M	0:10 (4°)	0:15 (52°)	2:00 (72°)	

* AccuTaq LA DNA polymerase; ** PrimeSTAR HS DNA polymerase

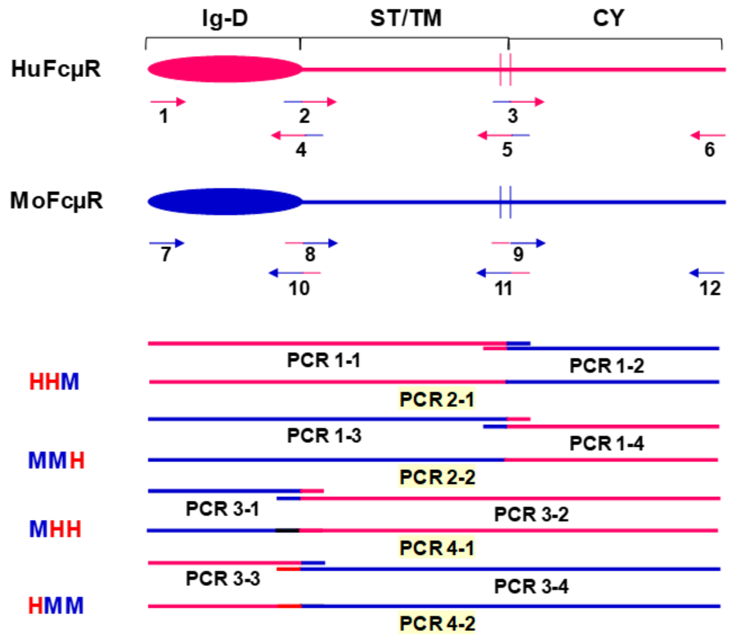

Figure S1: Strategy for overlap extension PCR for human/mouse chimeric FcμR constructs. Human (red) and mouse (blue) FcμRs are depicted as a racquet-like shape with the indicated functional domains, twelve oligonucleotide primers (arrows) and amplified PCR products (lines) according to the procedures indicated in Table S2. The final overlap extension PCR products [PCR 2-1 (HHM); PCR 2-2 (MMH); PCR 4-1 (MHH); PCR 4-2 (HMM)] are highlighted in yellow.
